# The Influence of Mat Pilates Training on Cardiometabolic Risk Factors in Postmenopausal Women with Single or Multiple Cardiometabolic Diseases

**DOI:** 10.3390/ijerph22010056

**Published:** 2025-01-02

**Authors:** Jaqueline Pontes Batista, Ana Luiza Amaral, Igor Moraes Mariano, Ludimila Ferreira Gonçalves, Julia Buiatte Tavares, Tállita Cristina Ferreira de Souza, Juliene Gonçalves Costa, Mateus de Lima Rodrigues, Jair Pereira da Cunha-Junior, Karine Canuto Loureiro de Araújo, Paula Aver Bretanha Ribeiro, Guilherme Morais Puga

**Affiliations:** 1 Laboratory of Cardiorespiratory and Metabolic Physiology, Physical Education and Physical Therapy Department, Federal University of Uberlândia, Uberlândia 38400-678, Brazil; jaquebpontes@cefetmg.br (J.P.B.); ana.amaral@ufu.br (A.L.A.); igor.mariano@ufu.br (I.M.M.); ludimila.goncalves@ufu.br (L.F.G.); juliabuiatte@ufu.br (J.B.T.); tallita.souza@ufu.br (T.C.F.d.S.); 2 School of Human Sciences (Exercise and Sport Science), University of Western Australia, Perth 6009, Australia; juliene.goncalvescostadechichi@research.uwa.edu.au; 3 Faculty of Electrical Engineering, Federal University of Uberlândia, Uberlândia 38400-902, Brazil; mateus.rodrigues@ufu.br; 4 Laboratory of Immunotechnology and Immunochemistry, Institute of Biomedical Sciences, Federal University of Uberlândia, Uberlândia 38400-902, Brazil; jair.cunha@ufu.br (J.P.d.C.-J.); karinecanuto@yahoo.com.br (K.C.L.d.A.); 5 Cardiovascular Health Across the Lifespan Program Research Institute of the McGill University Health Centre, Montreal, QC H4A 3J1, Canada; paula.ribeiro@rimuhc.ca

**Keywords:** menopause, exercise, chronic non-communicable diseases, lipids, adiponectin

## Abstract

This study compared the effects of Mat Pilates training on cardiovascular risk markers in postmenopausal women with single or multiple cardiometabolic conditions. Forty-four women were divided into single-condition (SINGLE; *n* = 20) and multiple-condition (MULTI; *n* = 24) groups. Both groups completed Mat Pilates three times per week for 12 weeks. Measurements of resting blood pressure, body composition, dietary intake, and blood markers were taken before and after the intervention. A Generalized Estimating Equation was used for hypothesis testing. MULTI presented higher body mass, BMI, fat mass, and waist circumference. Systolic blood pressure decreased more in SINGLE (−13 ± 15 mmHg) than in MULTI (−3 ± 16 mmHg, *p* interaction = 0.016 with diastolic reductions in both groups (SINGLE: −9 ± 12 mmHg; MULTI: −2 ± 11 mmHg, *p* interaction = 0.053). Triglycerides decreased only in SINGLE (−40 ± 98 mg/dL vs. +31 ± 70 mg/dL in MULTI, *p* interaction = 0.006), while no significant changes were observed in cholesterol levels. Adiponectin levels decreased in both groups (SINGLE: −1.5 ± 16.3; MULTI: −9.3 ± 12.4 vs. µg/dL, *p* time = 0.015). Glycated hemoglobin levels decreased over time in both groups (−0.3 ± 0.5% in SINGLE, −0.5 ± 0.6% in MULTI, *p* time < 0.001), with no significant changes in blood glucose. These findings suggest that Mat Pilates may be more effective in reducing cardiometabolic risk factors in women with a single condition compared to those with multiple conditions.

## 1. Introduction

The occurrence of multiple persistent conditions in a single patient, known as multimorbidity, has gained increasing attention during past decades [1]. In this context, women’s greater life expectancy puts them at higher risk for developing multiple chronic conditions, particularly cardiometabolic disorders (such as diabetes and heart disease) and obesity, leading to multimorbidity. Postmenopausal women are particularly vulnerable due to the reduction in estrogen production, which plays a key role in endothelial function, vascular tone, and cardiac function [2]. Consequently, the prevalence of hypertension is higher in postmenopausal women compared to their premenopausal counterparts [3,4]. Additionally, this population exhibits an increased incidence of dyslipidemia [5], abdominal obesity, and insulin resistance [6], all of which contribute significantly to the development of cardiometabolic diseases. Besides that, postmenopausal women often experience significant clinical symptoms that negatively impact their quality of life [7]. Therefore, preventive measures aimed at delaying or preventing the onset of new comorbidities should be prioritized, particularly in postmenopausal women [8,9].

In addition to factors related to hypoestrogenism and postmenopausal lifestyle, the development of other chronic conditions can be considered a complication of pre-existing diseases when left untreated. It is already well established by guidelines [5,10,11,12] how to treat chronic diseases independently, but there is still little evidence of the physiological responses of these associated diseases [13]. The likelihood of multimorbidity in women increases by 3% with each additional year of life, as well as with each increase in BMI [8,9]. In this sense, postmenopausal women are more prone to developing metabolic syndrome, as changes in weight and body fat distribution due to ovarian hormone deficiency contribute to insulin resistance, hypertension, and an adverse lipid profile [14,15]. Additionally, the duration of the chronic condition is associated with the likelihood of future comorbidities, meaning that individuals with a longer history of chronic illness have a higher probability of developing a second condition [16]. Beyond the rise in chronic diseases, multimorbidity can lead to a significant deterioration in health, reduced quality of life, increased healthcare costs, and a marked elevation in the risk of mortality with each additional disease [17].

On the other hand, non-pharmacological interventions, such as regular physical activity, are already recommended by guidelines to prevent multimorbidity, improve body composition, lipid profiles, glucose levels, and blood pressure (BP), and reduce cardiovascular risk and the development of cardiometabolic diseases [12,15,18]. The Pilates method is a versatile conditioning program aimed at enhancing strength, flexibility, and balance [19]. It can be practiced on specialized equipment or directly on the floor. Mat Pilates is the Pilates method without relying on traditional apparatus [20], incorporating resistance-based exercises with characteristics similar to bodyweight and functional fitness training. Accessories are often used to increase exercise intensity and load, offering a tailored approach to improve physical conditioning in diverse populations.

Resistance to physical exercise in postmenopausal women has been shown to be an important therapeutic approach to treating cardiometabolic diseases [21], improving bone mineral density [22] and maintenance/gain of muscle mass [23]. Furthermore, exercise interventions, including Mat Pilates, have been adopted by postmenopausal women [24]. Although Pilates is absent from the Fitness Trends list, related terms such as strength training with free weights and functional fitness training appear in the global Top 5 [25]. This method is a multicomponent intervention that has shown beneficial effects on cardiovascular, metabolic, and respiratory parameters in healthy individuals. Mat Pilates was designed to enhance strength, flexibility, balance, and mental conditioning, as it adheres to principles such as concentration, precision, fluid movement, and breath control [24,26].

In this scenario, while previous research has demonstrated the efficacy of Mat Pilates across various parameters such as muscle strength, lumbar pain, flexibility, and other muscle skeletal variables [7,27,28,29], its impact on cardiovascular and metabolic parameters such as blood pressure, lipid, and glycemic profile, and on body fat distribution remains unclear, particularly in postmenopausal women with multiple cardiometabolic conditions. Therefore, this study aimed to compare the effects of 12 weeks of Mat Pilates training on body composition, BP, lipid levels, glucose indices, and adiponectin in postmenopausal women with single and multiple cardiometabolic conditions. Our hypothesis was that the SINGLE group would exhibit better responses to Mat Pilates training compared to MULTI, as their physiological systems were more integrated and better equipped to adapt to physical exercise.

## 2. Materials and Methods

### 2.1. Participants

Participants were postmenopausal women (with at least 12 months of amenorrhea) aged between 50 and 70 years. Eligible volunteers had to meet the following criteria: no physical limitations affecting exercise performance, no use of hormonal therapies, and being non-smokers. Exclusion criteria included changes in medication type or dosage and participation in other physical activities alongside the intervention. All participants provided written informed consent and were assigned to one of the two groups described below. The study was approved by the local ethics committee of the Federal University of Uberlândia (68408116.9.0000.5152), adhered to the Helsinki Declaration, and was registered at ClinicalTrials.gov (NCT03626792).

A total of 806 women were recruited through traditional media (TV, radio, and posters) between March 2017 and March 2019, with 758 being excluded for not meeting the inclusion criteria. After the pre-intervention assessments, the remaining 48 participants began the program, which was divided into two groups based on the number of cardiometabolic chronic conditions: SINGLE—women with a single cardiometabolic condition, and MULTI—women with multimorbidity (two or more cardiometabolic conditions). In the present study, the following health conditions were considered [30]: obesity; diabetes; Mellitus; hypertension; dyslipidemia; history of stroke or cerebrovascular disease; and history of other heart or kidney diseases.

In the SINGLE group, one woman was excluded due to labyrinthitis, while two women withdrew for personal reasons, and one was lost to follow-up in the MULTI group due to increased antihypertensive dosage. In total, 44 postmenopausal women completed the study (SINGLE *n* = 20; MULTI *n* = 24). The recruitment process, participant characteristics, and training program followed the same protocols as described in our previous study [31].

### 2.2. General Procedures

All volunteers underwent body composition assessments, BP measurements, food recall, and blood sample collection before and after the Mat Pilates training period. Both pre- and post-tests were blinded to the researchers conducting the statistical analyses, and the researchers were also unaware of the participants’ group assignments during the training prescription. The same evaluators conducted all pre- and post-intervention tests. Post-tests were administered 72 h after the final training session.

### 2.3. Exercise Program

The Mat Pilates training program lasted for 12 weeks, with 3 sessions per week held for 50 min each. Each session included 5 min of warm-up, 40 min for the main workout, and 5 min for cooldown. A 45 s rest was provided between exercises. The main workout consisted of 20 classic Pilates exercises, selected based on a previous study (Supplementary Table S1, Supplement Digital Content S1, http://links.lww.com/BPMJ/A162 (accessed on 30 December 2024)) [32], and classified by the creator of the Pilates method. Only mats and alternative tools, such as the Swiss ball and flexible ring, were used. Bodyweight and gravity served as resistance factors, with Borg’s rating of perceived exertion scale (RPE) [33] used to monitor intensity.

Exercises were divided into two alternating sessions (A and B), incorporating variations in the same exercise, which targeted the same muscle groups, to ensure balanced training and allow progression while maintaining engagement. Intensity was controlled via RPE: 9 to 11 during warm-up and cooldown, and 11 to 15 during the main workout. Overload progression, previously described [31], was structured as follows: from weeks 1 to 3, participants performed 10 repetitions per exercise; from weeks 4 to 6, 12 repetitions; from weeks 7 to 9, ankle weights and dumbbells were introduced; and from weeks 10 to 12, 15 repetitions were performed. Throughout all exercises, participants were guided on breathing and body control. The 36 Mat Pilates sessions were scheduled on non-consecutive days, and if a participant missed a session, it was rescheduled. All Mat Pilates classes were taught by Physical Education professionals certified in the Pilates method.

### 2.4. Body Composition and Blood Pressure Measurements

Body composition and body mass were measured using the InBody 230 bioimpedance analyzer (Seoul, Republic of Korea). Height was measured with a fixed stadiometer (Sanny^®^, São Bernardo do Campo, SP, Brazil). Waist circumference was taken at the midpoint between the last rib and the iliac crest with an inelastic, flexible tape measure (Sanny, São Paulo, SP, Brazil), ensuring no compression of the skin. Body mass index (BMI) was calculated using the formula: BMI = weight (kg)/height^2^ (m).

Resting BP and heart rate (HR) were measured pre- and post-training in both groups using a validated and calibrated automatic oscillometric monitor (OMRON HEM-7113, Omron Healthcare Co., Ltd., Kyoto, Japan). On three non-consecutive days, before the intervention started and after the intervention ended, three BP and HR measurements were taken daily, and the mean values were used for analysis. All resting was standardized and conducted in the morning, by trained researchers, in the research laboratory, in a quiet place without third parties, in a seated position, after 20 min of rest in a quiet environment, with the back supported by the chair and the feet on the floor, with the arm supported at the height of the heart, as well as with the palm of the hand facing upwards and relaxed. The cuff was placed on the volunteer’s left arm, 2 to 3 fingers above the elbow joint.

### 2.5. Dietary Intake Assessment

All participants completed a 24 h food recall, in which we collected the recall for 3 days, conducted by trained nutritionists. They were asked to recall all foods and beverages consumed the previous day. Reports were collected for two non-consecutive weekdays and one weekend day, both at baseline and after 12 weeks of intervention. The purpose of the food recall was to determine whether the volunteers’ eating patterns would remain the same, since at the beginning of the study, we asked them to maintain their eating habits. Total energy intake, as well as protein, fat, carbohydrate, and fiber intake, were quantified using Dietpro^®^ software (version 5.7i, Agromídia Softwares^®^, Viçosa, Brazil) and the United States Department of Agriculture food composition table [34,35]. Additionally, manufacturers’ nutrition labels were also used for quantification.

### 2.6. Cardiometabolic Markers

The cardiometabolic markers were evaluated as described in the previous study [29]:

- obesity (IMC > 29.9 kg/m^2^ [10]);
- diabetes Mellitus (glycaemia > 126 mg/dL and/or HbA1c ≥ 6.5% [11]);
- hypertension or use of antihypertensive drugs (Resting systolic blood pressure—SBP > 139 mmHg, and Diastolic blood pressure—DBP > 89 mmHg [12]);
- dyslipidemia (LDL ≥ 160 mg/dL and/or triglycerides ≥ 150 mg/dL and/or total cholesterol ≥ 190 mg/dL and/or HDL ≤ 50 mg/dL [5]);
- history of stroke or cerebrovascular disease;
- history of other heart or kidney diseases.

### 2.7. Blood Samples Collection and Analysis

Fifteen mL blood samples were collected after an overnight fast, five days before and 72 h after the final exercise session, to mitigate the acute effects of exercise. These samples were placed in ethylenediaminetetraacetic acid (EDTA) or serum tubes with separator gel, depending on the assay, centrifuged at 3000 rpm for 15 min, and stored in microtubes for subsequent analysis. Total cholesterol, triglycerides (TG), high-density lipoprotein (HDL), low-density lipoprotein (LDL) cholesterol, and glucose concentrations were determined using enzymatic colorimetric methods with commercial kits (Labtest, Lagoa Santa, Brazil). Glycated hemoglobin concentrations were measured by turbidimetry. All analyses were conducted with an automated system (Cobas Mira, Roche Instruments Inc., Brookhaven, NY, USA), except for adiponectin, which was measured using a human ELISA kit (ELABSCIENCE, Wuhan, China).

### 2.8. Statistical Analysis

The sample size was calculated using G*Power 3.1 software, adopting a 5% α error, 80% statistical power, a correlation of 0.5 among repeated measures, and a nonsphericity correction of 1, in an F-family within-between analysis. Changes in BMI were considered the primary outcome. Based on the variations in BMI observed in a similar study with Mat Pilates intervention and population [36], the authors reported a Cohen’s d effect size of −0.44, which was converted to an effect size f of 0.22 for calculation purposes. Consequently, a minimum required sample size of 44 participants was determined.

Results are presented as mean ± standard deviation. Data characteristics were assessed using the Shapiro–Wilk normality test and the Levene test for homogeneity. A two-factor (time, group, and their interaction) Generalized Estimating Equation (GEE) was used for hypothesis testing (with a gamma distribution and log link for non-parametric data). Effect sizes were calculated as Cohen’s d [37]. Group baseline characteristics were compared using unpaired t-tests (or the Mann–Whitney test for non-parametric data). All analyses were performed using SPSS 26.0 software, with the level of significance set at *p* ≤ 0.05.

## 3. Results

There were no adverse effects from the training intervention, and all volunteers completed 36 sessions over 12 weeks on non-consecutive days, achieving 100% adherence. Table 1 presents the baseline general characteristics of both groups. Women in the MULTI group had higher BMI and TG levels at baseline compared to those in the SINGLE group, with no other significant differences observed.

Table 2 presents the body composition indices and resting SBP, DBP, and HR for both groups. There were no significant interaction effects for body composition indices. However, women in the MULTI group had higher body composition indices (body mass, BMI, fat mass, and waist circumference) compared to the SINGLE group (group effect). A significant interaction effect (*p* < 0.05) was observed only for SBP, which decreased exclusively in the SINGLE group. DBP decreased over time in both groups (time effect).

Table 3 presents the data on dietary intake. The groups started the intervention with similar dietary patterns, showing no significant differences at baseline (*p* > 0.05). Furthermore, no significant differences in dietary intake were observed during the intervention, indicating that the participants maintained their habitual dietary patterns.

Table 4 presents the blood sample analysis. A significant interaction effect was found for TG levels, which decreased exclusively in the SINGLE group. No other interaction effects were observed. Glycated hemoglobin levels decreased over time in both groups (time effect). Additionally, adiponectin levels were higher in the SINGLE group (group effect).

## 4. Discussion

Our study evaluated body composition, dietary intake, resting BP, lipid levels, glucose indices, and adiponectin responses in postmenopausal women with single or multiple cardiometabolic diseases after 12 weeks of Mat Pilates training. In summary, SBP and TG decreased only in women with single cardiometabolic condition, while DBP decreased in both groups after training, with a trend toward a greater reduction in the SINGLE group. Additionally, Mat Pilates training improved glycated hemoglobin levels in both groups but did not significantly affect body composition, cholesterol levels, glucose, or adiponectin, regardless of the number of comorbidities. Therefore, the initial hypothesis that the SINGLE group would exhibit better responses was confirmed.

In a previous study, we observed that hypertensive women exhibited higher SBP, DBP, and ambulatory sleep averages compared with normotensive women [32]. Additionally, in postmenopausal women, regular exercise training—particularly a combination of aerobic and resistance exercises—has been shown to effectively manage hypertension [38]. More recently, a study [39] demonstrated a significant reduction in SBP after 12 weeks of Mat Pilates training in hypertensive elderly individuals, which aligns with our findings. These results suggest that Mat Pilates can serve as an effective intervention for hypertension treatment, primarily through reducing SBP.

Building on these previous findings, our study showed even greater reductions in BP in the SINGLE group following Mat Pilates training. Unlike the study by Woramontri et al. (2024) [39], where elderly participants began training with higher baseline BP values, the BP values in our study did not differ significantly between groups at baseline. In the MULTI group, 83% of participants were hypertensive and on antihypertensive medication. As a result, their BP was already controlled, which may have limited further reductions through exercise, as their values were within normal or lower ranges. The regulatory mechanisms governing their BP likely prevented additional decreases, as their BP was already at optimal levels. Despite this, we believe the BP improvements observed in this study are meaningful for cardiovascular health in postmenopausal women, regardless of the number of cardiometabolic conditions they present.

In addition to cardiovascular improvements, metabolic parameters were also evaluated. In this regard, none of the volunteers in the present study had a diagnosis or were undergoing treatment for diabetes mellitus. This is further supported by the fact that the mean glucose and glycated hemoglobin levels were within the normal range in both groups [40]. The Mat Pilates training program did not significantly alter glucose levels but did reduce glycated hemoglobin levels in both groups. Consistent with our findings, low-volume resistance exercise in healthy post-menopausal women reduced glycated hemoglobin levels from slightly above the expected range (6%) to desirable levels [23]. Similarly, in elderly women with diabetes and elevated glycated hemoglobin levels (7.8%), a protocol comparable to ours resulted in a significant reduction (14%) [41]. These results, together with our findings, suggest that resistance-based exercises like Mat Pilates are effective in controlling glycated hemoglobin levels, regardless of the presence of cardiometabolic comorbidities.

Beyond glucose metabolism, lipid profile responses and fat mass were also analyzed. In this context, TG serve as the body’s primary energy reserve, stored in fat and muscle tissues [42]. Both blood cholesterol and TG abnormal levels may lead to cardiovascular metabolic disorders or diseases. The fat mass excess, particularly in the abdominal location, may lead to insulin resistance, atherosclerosis, endothelial dysfunction, and increase other cardiometabolic risk factors. Waist circumference and fat mass are important markers of fat accumulation and metabolic disorders, and both diet and physical exercise are important to improve and control it. Our study did not find any difference in fat mass or waist circumference, and we believe that exercise intensity and energy expenditure during the Mat Pilates method were not enough to improve it. It is important to note that caloric restriction is also important to reduce this parameter, but in our study, we did not change the death diet of the participants [8,42,43].

In this study, women in the SINGLE group began with TG levels slightly above the desirable range, but these levels reached the expected range after training. In contrast, the MULTI group showed no change in TG levels with training, but their values remained below the borderline for the undesired classification. The TG values in the SINGLE group appear to be lower after the intervention, and these changes could be due to acute natural changes in TG or changes in fat ingestion in the days before the blood collection. The 24 h food recall did not show a difference in lipids intake between groups or periods of time, so these lower TG values could be due to natural variation or other causes not measured in the study. It is important to note that no statistical interactions were found in the TG responses between groups. Although physical exercise is typically effective in reducing TG levels, this effect can diminish in individuals over 50 years of age [43]. In a study involving postmenopausal women [44], Pilates training successfully reduced TG, even in overweight and obese participants, though the values did not significantly differ from the control group without exercise, as both groups maintained TG levels within the desired range throughout the protocol. Therefore, while we observed a statistically significant difference, clinically, the TG levels did not fall into undesirable ranges. So, we believe that the reduction in the SINGLE group is likely attributable to their higher baseline TG levels compared to the MULTI group.

Complementing the lipid profile and fat mass analysis, we also investigated Adiponectin, as it is a hormone primarily secreted by adipose tissue, playing a key role in fatty acid oxidation, glycemic regulation, and anti-inflammatory activity [45]. The Adiponectin levels are inversely correlated with fat mass, insulin resistance and glucose tolerance, and chronic lower-grade inflammation levels [46]. The changes in Adiponectin levels after exercise may contribute to improving lipid profile, by activation of AMPK, which plays an important role in energy metabolism and lipid oxidation. In this study, the SINGLE group demonstrated higher serum levels of adiponectin, suggesting that women with fewer comorbidities and lower waist circumference exhibit higher concentrations of this hormone. Conversely, in obese postmenopausal women, the lower adiponectin levels observed in the MULTI group indicate a greater risk of developing metabolic syndrome [46]. However, higher adiponectin levels do not appear to offer a protective effect in this population [46], leading us to suggest that obesity may inhibit some of the favorable effects of adiponectin. A meta-analysis [47], found that aerobic exercise could increase adiponectin levels in overweight and obese individuals, while resistance-based exercise had no significant effect on these levels, consistent with our findings. Therefore, Mat Pilates may not be the most effective method for increasing adiponectin levels.

In the present study, dietary assessments revealed no significant differences in caloric intake over time or between groups, indicating that body composition was not influenced by diet. In this context, the effects of Mat Pilates on body composition remain controversial. Improvements in fat percentage and muscle mass have been reported in adult women [48] and reductions in fat percentage and fat mass have been observed in elderly women [24]. Additionally, combined aquatic aerobic exercises and Mat Pilates training have shown improvements in anthropometric measures [49]. However, consistent with the findings of the present study, Bergamin et al. [50] did not demonstrate changes in body mass following Mat Pilates training in postmenopausal women.

We acknowledge some limitations in the present study, such as the relatively short intervention period (12 weeks), which may have been insufficient to induce significant anthropometric and metabolic changes. Additionally, we did not include more complex cases of multimorbidity, such as heart disease, severe obesity, or individuals with more than three comorbidities. Furthermore, a control group without exercise was not included, although the study’s aim was to compare exercise-induced responses between women with and without multimorbidity. It is also important to note that the women in the MULTI group were under well-controlled conditions through medication, which may limit the generalizability of these results to populations with less effective or no treatment. Moreover, the use of the 24 h dietary recall presents inherent limitations, particularly in populations with obesity. These include underreporting of dietary intake, which may be influenced by social desirability bias or memory recall issues, as well as difficulties in estimating portion sizes accurately. These factors could affect the precision of the dietary data collected.

Despite these limitations, we emphasize that few studies provide the level of detail in Mat Pilates training periodization as ours, which enhances the reproducibility and clinical applicability of the results. Additionally, although the effects did not differ significantly between groups, we believe that all postmenopausal women could benefit from the cardiometabolic improvements observed in this study, such as reductions in BP and glycated hemoglobin.

For future research, we suggest that studies be conducted with different populations and incorporate other forms of Pilates, such as equipment-based Pilates training. We also emphasize that for greater effects, the inclusion of additional modalities, such as aerobic training, should be considered. Adopting broader healthy lifestyle habits—including a appropriate diet, regular physical activity, and abstinence from alcohol and tobacco—would further enhance outcomes. Additionally, combining Mat Pilates with aerobic exercise could help reduce the monotony of physical activity and potentially yield more significant cardiometabolic benefits. These future directions could help expand upon our findings that Mat Pilates can effectively improve cardiometabolic health in postmenopausal women, particularly those with single cardiometabolic disease.

The clinical implications of our study suggest that Mat Pilates can be a valuable non-pharmacological intervention for improving specific cardiometabolic health markers in postmenopausal women, particularly those with a single cardiometabolic condition. Clinically, Mat Pilates could be incorporated into routine care for postmenopausal women as a low-impact, accessible exercise modality that supports cardiovascular health and glycemic control, complementing pharmacological treatments and other lifestyle interventions such as dietary modifications and aerobic exercise. Further, its structured and periodized nature enhances its practicality for clinical settings, making it a feasible option for the long-term management of cardiometabolic health in aging women.

## 5. Conclusions

In conclusion, blood pressure improved more significantly, and triglycerides improved only in women with single cardiometabolic disease after twelve weeks of Mat Pilates training. Additionally, both blood pressure and glycated hemoglobin showed improvements in both groups, regardless of the number of comorbidities in postmenopausal women. This training did not lead to changes in anthropometric parameters, lipid profiles, adiponectin levels, or glycemia. In summary, Mat Pilates can be a safe and effective method for managing and preventing cardiometabolic risk factors in postmenopausal women, especially triglycerides, blood pressure, and glycated hemoglobin.

## Figures and Tables

**Table 1:** Baseline general characteristics and dietary intake assessment in “mean ± standard deviation” or “*n* (%)”.

	SINGLE	MULTI	Overall
	*n* = 20	*n* = 24	*n* = 44
Characteristics			
Age (years)	57 ± 4	58 ± 6	58 ± 5
Time after menopause (years)	10 ± 6	9 ± 7	9 ± 7
BMI (kg/m^2^)	25 ± 3	29 ± 4 ^#^	27 ± 3
BMI > 29.9 (kg/m^2^)	0 (0)	11 (46)	11 (25)
25.0 < BMI < 29.9 (kg/m^2^)	10 (50)	9 (24)	19 (43)
BMI < 24.9 (kg/m^2^)	10 (50)	4 (17)	14 (32)
Resting SBP (mmHg)	121 ± 8	119 ± 10	120 ± 9
Resting DBP (mmHg)	77 ± 8	76 ± 8	77 ± 8
Hypertension	3 (15)	20 (83)	23 (52)
Fasting Glucose (mg/dL)	97 ± 10	93 ± 8	95 ± 9
Fasting Glucose > 126 (mg/dL)	0 (0)	0 (0)	0 (0)
Total Cholesterol (mg/dL)	231 ± 40	218 ± 35	224 ± 37
Total Cholesterol > 190 (mg/dL)	17 (85)	21 (88)	38 (86)
HDL (mg/dL)	60 ± 11	61 ± 11	61 ± 11
HDL < 50 (mg/dL)	3 (15)	20 (83)	23 (52)
LDL (mg/dL)	142 ± 39	135 ± 33	139 ± 36
LDL > 130 (mg/dL)	13 (65)	13 (54)	26 (59)
Triglycerides (mg/dL)	162 ± 89	113 ± 44 ^#^	138 ± 67
Triglycerides > 150 (mg/dL)	8 (40)	5 (21)	13 (30)
Number of comorbidities (*n* (%))			
One	20 (100)	0 (0)	20 (45)
Two	0 (0)	20 (83)	20 (45)
Three	0 (0)	4 (17)	4 (9)
Four or more	0 (0)	0 (0)	0 (0)
Medications (*n* (%))			
Statins	1 (5)	1(4)	2 (5)
ARB	1 (5)	6 (5)	7 (16)
ARB + Diuretic	2 (10)	7 (29)	9 (20)
Diuretic	0 (0)	2 (9)	2 (5)
ACE inhibitor	0 (0)	4 (17)	4 (9)
ACE inhibitor + Diuretic	0 (0)	1 (4)	1 (32
Levothyroxine	3 (15)	3 (13)	6 (14)

SINGLE: women with 1 cardiometabolic disease; MULTI: women with ≥2 cardiometabolic diseases; BMI: body mass index; HDL: high density lipoprotein; LDL low density lipoprotein; ARB: Angiotensin receptor blocker; ACE: Angiotensin-converting enzyme. ^#^ *p* < 0.05 in *t* test comparing groups.

**Table 2:** Body composition and resting blood pressure and heart rate pre- and post-12 weeks of Mat Pilates exercise training.

	Pre	Post	∆	*p*	*p*	*p*	Cohen’s d
	Mean ± SD	Mean ± SD	Mean ± SD	Group	Time	Group × Time	
Body mass (kg)							
SINGLE	64.1 ± 7.1	63.6 ± 6.9	−0.4 ± 1.9	<0.001	0.970	0.684	0.07
MULTI	71.8 ± 8.5	72.2 ± 8.3	0.4 ± 1.1				0.05
Body mass index (kg/m^2^)							
SINGLE	25 ± 3	25 ± 2	−0.2 ± 0.8	<0.001	0.968	0.690	0.00
MULTI	29 ± 4	29 ± 4	0.2 ± 0.7				0.00
Fat mass (kg)							
SINGLE	22.9 ± 5.1	22.8 ± 4.6	−0.2 ± 1.6	<0.001	0.830	0.665	0.02
MULTI	29.4 ± 7.4	30.0 ± 7.0	0.6 ± 1.9				0.08
Waist circumference (cm)							
SINGLE	89.9 ± 6.1	87.9 ± 6.3	−1.1 ± 5.5	<0.001	0.328	0.955	0.32
MULTI	95.8 ± 8.9	94.5 ± 7.1	−1.3 ± 6.8				0.16
Systolic blood pressure (mmHg)							
SINGLE	121 ± 8	108 ± 11	−13 ± 15	0.106	<0.001	0.016	1.35
MULTI	119 ± 10	116 ± 8	−3 ± 16				0.33
Diastolic blood pressure (mmHg)							
SINGLE	77 ± 8	69 ± 8	−9 ± 12	0.119	0.001	0.053	1.00
MULTI	76 ± 8	74 ± 8	−2 ± 11				0.25
Resting Heart Rate (bpm)							
SINGLE	71 ± 8	67 ± 8	−4 ± 12	0.033	0.181	0.401	0.50
MULTI	73 ± 9	72 ± 11	−1 ± 12				0.10

SINGLE: women with 1 cardiometabolic disease (*n* = 20); MULTI: women with ≥2 cardiometabolic diseases (*n* = 24)**.**

**Table 3:** Dietary intake characteristics pre- and post-12 weeks of Mat Pilates exercise training.

	Pre	Post	∆	*p*	*p*	*p*	Cohen’s d
	Mean ± SD	Mean ± SD	Mean ± SD	Group	Time	Group × Time	
Energy (kcal)							
SINGLE	1653.02 ± 478.67	1593.98 ± 428.47	−59 ± 276	0.913	0.555	0.738	0.13
MULTI	1647.76 ± 587.1	1631.25 ± 469.1	−93 ± 684				0.03
Protein (g)							
SINGLE	62.84 ± 14.43	58.79 ± 17.53	−4 ± 13	0.141	0.108	0.667	0.25
MULTI	74.36 ± 36.08	66.27 ± 22.6	−11 ± 34				0.27
Lipids (g)							
SINGLE	71.25 ± 27.36	69.52 ± 20.67	−2 ± 15	0.921	0.193	0.357	0.07
MULTI	76.41 ± 38.06	66.26 ± 24.05	−14 ± 38				0.32
Saturated lipids (g)							
SINGLE	23.18 ± 11.26	19.79 ± 6.73	−3 ± 9	0.529	0.08	0.774	0.37
MULTI	21.13 ± 8.71	18.87 ± 7.25	−3 ± 11				0.28
Monoinsaturated lipids (g)							
SINGLE	19.5 ± 7.74	17.59 ± 5.13	−2 ± 5	0.552	0.156	0.929	0.29
MULTI	20.71 ± 9.78	18.9 ± 7.98	−3 ± 11				0.20
Polinsaturated lipids (g)							
SINGLE	18.86 ± 7.15	19.92 ± 7.57	1 ± 5	0.729	0.912	0.392	0.14
MULTI	18.97 ± 8.63	18.18 ± 7.94	−1 ± 8				0.09
Cholesterol (mg)							
SINGLE	273.65 ± 140.03	235.56 ± 98.08	−38 ± 89	0.37	0.051	0.976	0.32
MULTI	243.65 ± 118.05	208.75 ± 90.24	−40 ± 149				0.33
Carbohydrates (g)							
SINGLE	192.84 ± 52.95	184.15 ± 51.87	−9 ± 44	0.943	0.855	0.201	0.17
MULTI	181.56 ± 57.53	193.08 ± 64.85	5 ± 84				0.19
Fiber (g)							
SINGLE	15.92 ± 4.09	15.52 ± 5.74	0 ± 3	0.734	0.856	0.430	0.08
MULTI	14.83 ± 5.58	15.44 ± 6.82	1 ± 8				0.10

SINGLE: women with 1 cardiometabolic disease (*n* = 20); MULTI: women with ≥2 cardiometabolic diseases (*n* = 24).

**Table 4:** Lipid profile, glucose indexes, and adiponectin plasma levels pre- and post-12 weeks of Mat Pilates exercise training.

	Pre	Post	∆	*p*	*p*	*p*	Cohen’s d
	Mean ± SD	Mean ± SD	Mean ± SD	Group	Time	Group × Time	
Total cholesterol (mg/dL)							
SINGLE	231 ± 40	222 ± 43	−9 ± 45	0.178	0.194	0.975	0.22
MULTI	218 ± 35	210 ± 31	−8 ± 52				0.24
HDL (mg/dL)							
SINGLE	60 ± 11	63 ± 12	3 ± 12	0.387	0.882	0.092	0.26
MULTI	61 ± 11	58 ± 12	−4 ± 17				0.26
LDL (mg/dL)							
SINGLE	142 ± 39	135 ± 40	−7 ± 42	0.310	0.199	0.796	0.18
MULTI	135 ± 33	126 ± 28	−10 ± 50				0.29
Triglycerides (mg/dL)							
SINGLE	161 ± 89	122 ± 57	−40 ± 98	0.390	0.728	0.006	0.57
MULTI	113 ± 44	144 ± 60	31 ± 70				0.59
Glucose (mg/dL)							
SINGLE	97 ± 10	93 ± 8	−4 ± 14	0.418	0.628	0.218	0.44
MULTI	93 ± 8	94 ± 9	2 ± 14				0.12
Glycated hemoglobin (%)							
SINGLE	5.6 ± 0.2	5.3 ± 0.5	−0.3 ± 0.5	0.068	<0.001	0.303	0.39
MULTI	5.5 ± 0.3	5.1 ± 0.5	−0.5 ± 0.6				0.97
Adiponectin (µg/dL)							
SINGLE	34.4 ± 13.3	32.8 ± 18.0	−1.5 ± 16.3	0.034	0.015	0.012	0.10
MULTI	28.5 ± 16.0	19.2 ± 12.8	−9.3 ± 12.4				0.64

SINGLE: women with 1 cardiometabolic disease (*n* = 20); MULTI: women with ≥ 2 cardiometabolic diseases (*n* = 24); HDL: high density lipoprotein; LDL: low density lipoprotein.

## Data Availability

The data that support the findings of this study are available on request from the corresponding author GMP.
